# The effects of macrophages on cardiomyocyte calcium‐handling function using in vitro culture models

**DOI:** 10.14814/phy2.14137

**Published:** 2019-07-12

**Authors:** Pamela G. Hitscherich, Lai‐Hua Xie, Dominic Del Re, Eun Jung Lee

**Affiliations:** ^1^ Department of Biomedical Engineering New Jersey Institute of Technology Newark New Jersey; ^2^ Department of Cell Biology and Molecular Medicine Rutgers New Jersey Medical School Newark New Jersey

**Keywords:** Cardiomyocyte, inflammation, macrophage, matricellular protein, pluripotent stem cells

## Abstract

Following myocardial infarction (MI), myocardial inflammation plays a crucial role in the pathogenesis of MI injury and macrophages are among the key cells activated during the initial phases of the host response regulating the healing process. While macrophages have emerged as attractive effectors in tissue injury and repair, the contribution of macrophages on cardiac cell function and survival is not fully understood due to complexity of the in vivo inflammatory microenvironment. Understanding the key cells involved and how they communicate with one another is of paramount importance for the development of effective clinical treatments. Here, novel in vitro myocardial inflammation models were developed to examine how both direct and indirect interactions with polarized macrophage subsets present in the post‐MI microenvironment affect cardiomyocyte function. The indirect model using conditioned medium from polarized macrophage subsets allowed examination of the effects of macrophage‐derived factors on stem cell‐derived cardiomyocyte function for up to 3 days. The results from the indirect model demonstrated that pro‐inflammatory macrophage‐derived factors led to a significant downregulation of cardiac troponin T (cTnT) and sarcoplasmic/endoplasmic reticulum calcium ATPase (Serca2) gene expression. It also demonstrated that inhibition of macrophage‐secreted matricellular protein, osteopontin (OPN), led to a significant decrease in cardiomyocyte store‐operated calcium entry (SOCE). In the direct model, stem cell‐derived cardiomyocytes were co‐cultured with polarized macrophage subsets for up to 3 days. It was demonstrated that anti‐inflammatory macrophages significantly increased cardiomyocyte Ca^2+^ fractional release while macrophages independent of their subtypes led to significant downregulation of SOCE response in cardiomyocytes. This study describes simplified and controlled in vitro myocardial inflammation models, which allow examination of potential beneficial and deleterious effects of macrophages on cardiomyocytes and vise versa. This can lead to our improved understanding of the inflammatory microenvironment post‐MI, otherwise difficult to directly investigate in vivo or by using currently available in vitro models.

## Introduction

Each year, millions of people suffer from myocardial infarction (MI) in the Western world (Benjamin et al. 2017). Even those patients who survive MI are left with irreversible damage to the heart and changes in ventricle geometry that result in decreased cardiac performance (Pfeffer and Braunwald 1990). Despite the immense significance, little is still known about the specific roles of key cell types involved in post‐MI myocardial survival, remodeling and function. Macrophages are one of the key cell types involved during the inflammation phase post‐MI (Dewald et al. 2005; Prabhu and Frangogiannis 2016). It is now recognized that there is a complex spectrum of macrophage phenotypes in the post‐MI microenvironment. Macrophages with a pro‐inflammatory phenotype, described generally as M1 type, are involved in 1–3 days post‐MI. More anti‐inflammatory or pro‐healing phenotypes of macrophages, described generally as M2 type, are involved in days 5–7 post‐MI (Nahrendorf et al. 2007; Troidl et al. 2009; Swirski and Nahrendorf 2013; Honold and Nahrendorf 2018).

Both the pro‐ and anti‐inflammatory macrophage subsets secrete an array of cytokines that contribute to the post‐MI microenvironment. One class of proteins secreted by macrophages after injury is matricellular proteins (Frangogiannis 2012). Matricellular proteins are nonstructural extracellular matrix proteins, however not much detail is known about their effect on different cell types, including cardiomyocytes. One matricellular protein of interest known to be involved with inflammation through constitutive expression and secretion by macrophages is osteopontin (OPN) (Murry et al. 1994). It is highly upregulated at the infarct border zone post‐MI (Murry et al. 1994) and has been associated with the development of cardiac disease, specifically cardiac hypertrophy and heart failure (Graf et al. 1997; Stawowy et al. 2002; Xie et al. 2004). However, the extent of OPN involvement in the post‐MI microenvironment and how OPN is linked to cardiac disease post‐MI is not understood despite it being previously used as a predictor of adverse outcomes related to vascular conditions (Lutz et al. 2017) and mortality in patients with chronic heart failure (Rosenberg et al. 2008). Hence, to better understand this complex inflammatory microenvironment post‐MI, there is a need for a well‐controlled in vitro disease model that can serve as a powerful tool to evaluate the heterocellular cross talk during post‐MI repair process.

Very recently, Ai et al. examined the effect of lipopolysaccharide (LPS)‐treated pro‐inflammatory macrophages on the survival of rat H9c2 cells using a microfluidic device. They demonstrated increased myocyte apoptosis via mitochondrial damage when in co‐culture with LPS‐treated pro‐inflammatory macrophages (Ai et al. 2018). However, phenotypic or functional changes of myocytes were not examined. In addition, the use of only pro‐inflammatory macrophages limited examination of the influence of temporally changing macrophage phenotypes present in the post‐MI microenvironment. Another study demonstrated a noticeable improvement in cardiac function and a decrease in mortality and infarct size with the inhibition of macrophage infiltration and pro‐inflammatory cytokine production (Ji et al. 2018). In addition, Tokutome et al. used nanoparticles (NPs) loaded with an anti‐inflammatory drug to inhibit monocyte recruitment and inflammatory gene expression in macrophages. NPs lessened ischemia‐reperfusion injury and significantly reduced mouse mortality (Tokutome et al. 2019). Despite these recent studies, details regarding macrophage subpopulations and their effect on resident cardiomyocytes or on other cell types administered during cell‐based therapy remain unclear.

In this study, two in vitro myocardial inflammation models were created to better understand the interaction between macrophages and cardiomyocytes during the inflammatory phase post‐MI. The first model used macrophage‐conditioned medium to culture pluripotent stem cell‐derived cardiomyocytes. The second model allows direct co‐culture of polarized macrophage subsets with pluripotent stem cell‐derived cardiomyocytes. These models permit detailed characterization of gene expression by macrophages and cardiomyocytes, expression and secretion of cytokines and more importantly, the effects macrophages or macrophage‐derived factors have on the calcium‐handling properties of cardiomyocytes, which is otherwise difficult to examine in vivo.

## Materials and Methods

### Mouse embryonic stem (mES) cell culture and differentiation

A stable cardiac troponinT‐eGFP mES cell line (Lee et al. 2011) was differentiated into cardiomyocytes (mES‐CM) via hanging drops and a discontinuous Percoll purification technique as described in detail by our group (Hitscherich et al. 2016, 2018). No mycoplasma testing was performed.

### Macrophage culture and polarization

A RAW 264.7 macrophage cell line (Ralph and Nakoinz 1977) was maintained in an unpolarized state in RPMI medium supplemented with 10% fetal bovine serum (Atlanta Biologics) and 1% penicillin/streptomycin (Gibco). Macrophages were then serum starved for 24 h followed by either lipopolysaccharides (LPS, 100 ng/mL, Sigma) or IL‐4 (20 ng/mL, Biolegend) supplementation for 24 h for polarization toward either a pro‐ or anti‐inflammatory phenotype, respectively (Martinez and Gordon 2014). No mycoplasma testing was performed.

### Indirect myocardial inflammation model

**Figure 1:**
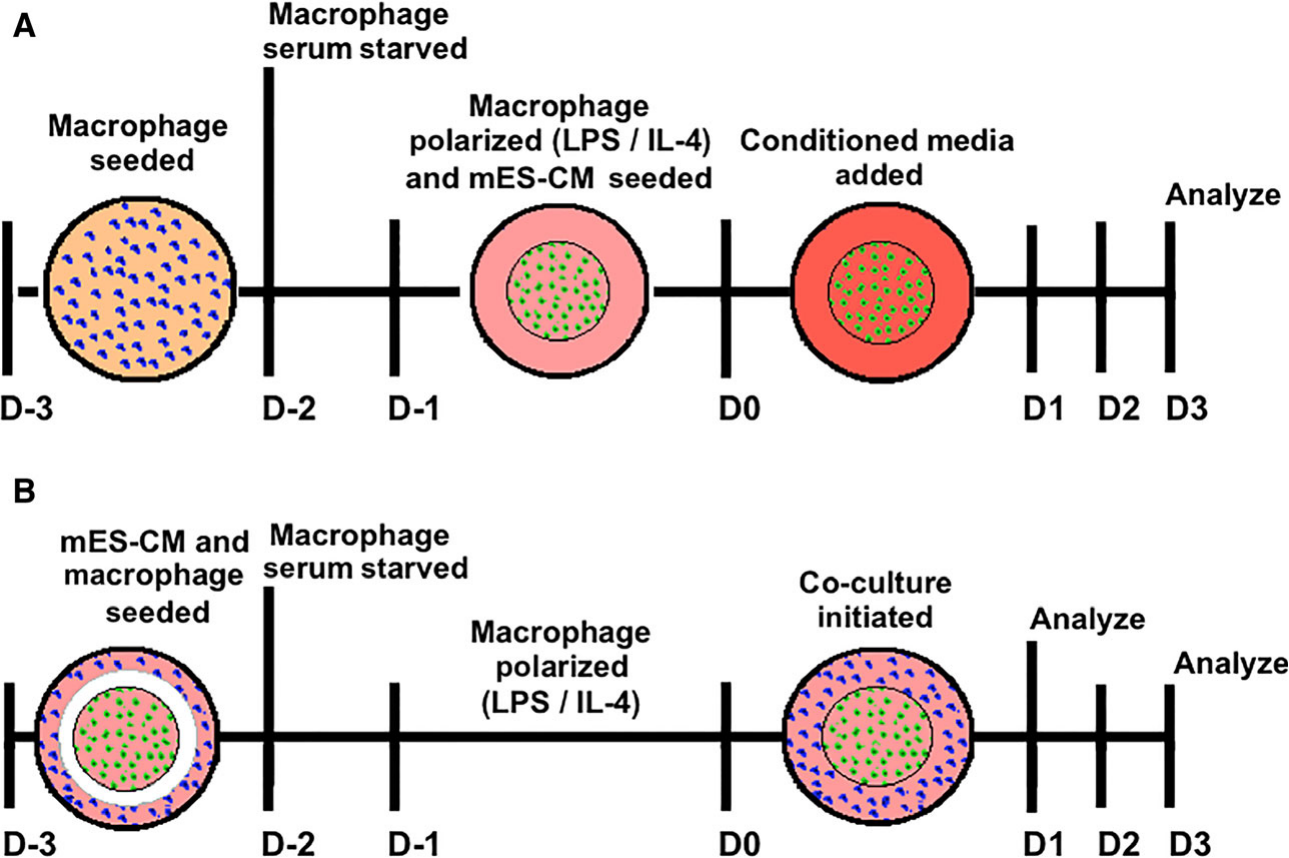
Schematic of an (A) indirect and (B) direct co‐culture of macrophages and cardiomyocytes.

A schematic of the indirect co‐culture model is shown in Figure 1A. MES‐CM (250,000 cells) were seeded on the glass center of the glass bottom culture dish (MatTek). Macrophages were cultured in a 6‐well plate (40,000 cells/well) and polarized as described above. Conditioned medium from macrophage cultures was then centrifuged (3 min, 10,000 RPM) to remove any debris or cells before mixing in 1:1 ratio with cardiomyocyte differentiation media to culture mES‐CM for up to 72 h.

### Direct co‐culture myocardial inflammation model

A schematic of the direct co‐culture model is shown in Figure 1B. Initially 20,000 macrophages are seeded in the outer edges of a glass bottom dish (MatTek) in RPMI‐supplemented media while 250,000 mES‐CM are seeded in the glass center region of the dish in differentiation media. The following day, macrophage medium is replaced with serum‐free medium for 24 h and then with either serum‐containing media or LPS or IL‐4 supplemented media for another 24 h for polarization. Culture medium of macrophage and cardiomyocyte was then mixed in a 1:1 ratio to initiate the co‐culture. Samples were analyzed either after 24 or 72 h.

### RT‐PCR

Total RNA was extracted and purified from mES‐CM and macrophages using the GenElute Mammalian Total RNA Miniprep Kit following the manufacturer's instruction (Fisher Scientific). cDNA was created with 500 ng of RNA and the High‐Capacity cDNA Reverse Transcription Kit (Applied Biosystems) in the T100 Thermal Cycler (Bio‐rad). RT‐PCR reactions were then prepared with the SSo Advanced SYBRgreen Mastermix (Bio‐rad). The primers used in the study are listed in Table 1 (IDT) (Schroeder et al. 2006; Poh et al. 2014). The qPCR reaction was then run in a CFX Connect Real Time System (Bio‐rad) for up to 50 cycles. Changes in the gene expression were presented using the Comparative C_T_ method as described previously (Schmittgen and Livak 2008).

**Table 1:** RT‐PCR primer list

Target	Forward	Reverse
cTnT	GAGACAGAGGAGGCCAACGTA	CTTTCCTTCTCCCGCTCAT
Serca2	TGCCTGGTAGAGAAGATGAA	CCCTTCACAAACATCTTGCT
My6	CTCCTTGTCATCAGGCAC	ACATTCTTCAGGATTCTCTG
My7	CTTCTCAGACTTCCGCAG	TTCCTTACTTGCTACCCTC
Bax	TGCAGAGGATGATTGCTGAC	GATCAGCTCGGGCACTTTAG
BCL‐2	ACAACCGCGAGCCAGGTA	CAGGGCATAGAACTCGGAAG
TNF‐*α*	CGTCAGCCGATTTGCTATCT	CGGACTCCGCAAAGTCTAAG
IL‐1*β*	GCCCATCCTCTGTGACTCAT	AGGCCACAGGTATTTTGTCG
Nos2	GAGCGAGGAGCAGGTGGAAGACTA	GCGCTGCCCTTTTTTGCCCCATAG
Cox2	TTCACCCGAGGACTGGGCCATGGA	GCCCCACAGCAAACTGCAGGTTCT
YM1	GAAGCCCTCCTAAGGACAAAC	GCAGCCTTGGAATGTCTTTCT
Arg1	AGGCCCTGCAGCACTGAGGAA	GCCAGGTCCCCGTGGTCTCTCA
*β*‐actin	GAT CTG GCA CCA CAC CTT CT	GGG GTG TTG AAG GTC TCA AA

### Immunofluorescence imaging

The use of a stable cardiac troponinT‐eGFP mES cell line allowed examination of cardiac troponin T expression by mES‐CM during culture with a confocal fluorescence microscope (IX81 DSO, Olympus). Macrophages in monoculture were fixed overnight using 4% formaldehyde (Sigma Aldrich) after 24 h of polarization. Samples were stained for F‐actin using Rhodamine‐conjugated phalloidin (1:20, Life Technologies) for 2 h at room temperature and counter‐stained with DAPI to visualize nuclei before imaging with a confocal fluorescence microscope (IX81 DSU, Olympus) (Hitscherich et al. 2016, 2018).

### Protein secretion

A cytokine array (R&D Systems) was used to examine the secretion of inflammatory cytokines in culture following manufacturer's instructions. Culture medium was collected from both polarized macrophages and cardiomyocytes in monocultures or from co‐culture media after 72 h. Secretion of OPN by macrophages, mES‐CM and co‐culture after 72 h was quantified using a Mouse/Rat osteopontin Quantikine ELISA (R&D Systems) following manufacturer's instructions.

### OPN inhibition and addition

In the indirect model, conditioned medium was collected from polarized macrophages and mixed with mES‐CM differentiation media in 1:1 ratio. OPN‐specific antibody was added (100 *μ*g/mL, R&D Systems) to make a final concentration of 6 *μ*g/mL. MES‐CM were then cultured in this OPN inhibition media for 3 days. For addition of OPN in culture, recombinant mouse OPN (R & D Systems) was added to mES‐CM in monoculture yielding a final working concentration of 5 *μ*g/mL.

### Evaluation of calcium (Ca^2+^)‐handling properties

After 24 or 72 h, mES‐CM seeded in glass bottom dishes were assessed for calcium‐handling properties as previously described by our group (Hitscherich et al. 2016, 2018). Additionally, for the exploration of SOCE, mES‐CM were challenged with 10 mmol/L Caffeine (Caff) along with 10 *μ*mol/L cyclopiazonic acid (CPA, Sigma Aldrich), a potent sarco/endoplasmic reticulum ATPase inhibitor (Wen et al. 2018).

### Statistical analysis

Results are presented as mean ± standard deviation or as individual data points and an average. Statistical analysis was performed in SPSS (Version 24, IBM) using a two‐tailed independent samples *t*‐test, one‐way ANOVA or their nonparametric counterpart, followed by Tukey and Games‐Howell post hoc tests, where appropriate. Statistical significance was accepted for *P < 0.05.

## Results

### Monoculture macrophage phenotype characterization

**Figure 2:**
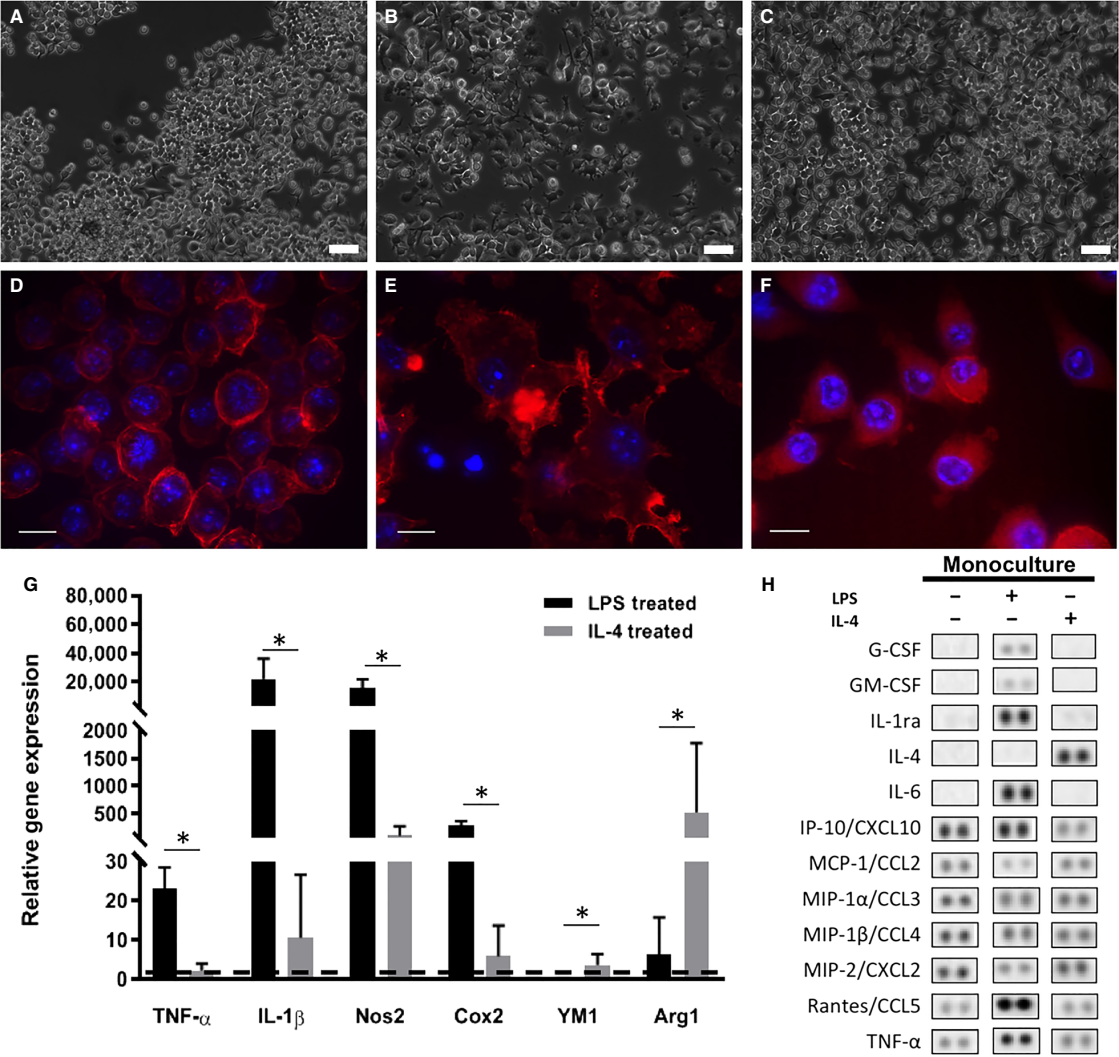
(A) Raw264.7 macrophages were polarized into (B) a pro‐inflammatory phenotype with LPS treatment or into (C) an anti‐inflammatory phenotype with IL‐4 treatment (scale bars = 50 *μ*m). F‐actin staining with rhodamine‐conjugated phalloidin (red) demonstrates the morphological features of (D) untreated, (E) LPS‐treated or (F) IL‐4‐treated macrophages (scale bars = 10 *μ*m). (G) Gene expression profiles of LPS‐ and IL‐4‐treated macrophages after 24 h of activation relative to untreated macrophages (LPS‐treated *n* = 7, Il‐4‐treated *n* = 6, Independent Samples *T*‐test or Mann–Whitney Test, *P < 0.05). (H) Inflammatory cytokine secretion from untreated, LPS and IL‐4‐treated macrophages in monoculture after 3 days (*n* = 1).

Macrophages (Fig. 2A and D) were successfully polarized into either a pro‐inflammatory phenotype with treatment of LPS or an anti‐inflammatory phenotype with IL‐4. LPS‐treated macrophages demonstrated a more spread out morphology with numerous projections (Fig. 2B and E) while IL‐4‐treated cells retained a rounded morphology (Fig. 2C and F) similar to untreated controls. LPS‐treated macrophages demonstrated significantly higher expression of pro‐inflammatory genes such as TNF‐*α*, IL‐1*β*, Cox2 and Nos2 compared to IL‐4‐treated macrophages (*P* < 0.05). IL‐4‐treated macrophages expressed significantly higher levels of anti‐inflammatory genes such as YM1 and ARG1 compared to LPS‐treated macrophages (*P* < 0.05, Fig. 2G). Additionally, LPS treatment for 3 days induced secretion of pro‐inflammatory cytokines including granulocyte colony‐stimulating factor (G‐CSF), granulocyte‐macrophage colony‐stimulating factor (GM‐CSF), interleukin‐1 receptor agonist (IL‐1ra), interleukin‐6 (IL‐6), regulated on activation normal T cell expressed and secreted (RANTES) and tumor necrosis factor alpha (TNF‐*α*) (Fig. 2H). Conversely, IL‐4 treatment downregulated the secretion of pro‐inflammatory cytokines such as G‐CSF, GM‐CSF, IL‐1ra, IL‐6, interferon gamma‐induced protein (IP‐10), RANTES and TNF‐*α* and upregulated IL‐4 secretion (Fig. 2H). Expression of monocyte chemoattractant protein 1 (MCP‐1), macrophage inflammatory protein 2 (MIP‐2), MIP‐1*α* and MIP‐1*β* was similar among groups (Fig. 2H).

### Indirect myocardial inflammation model: mES‐CM characterization

**Figure 3:**
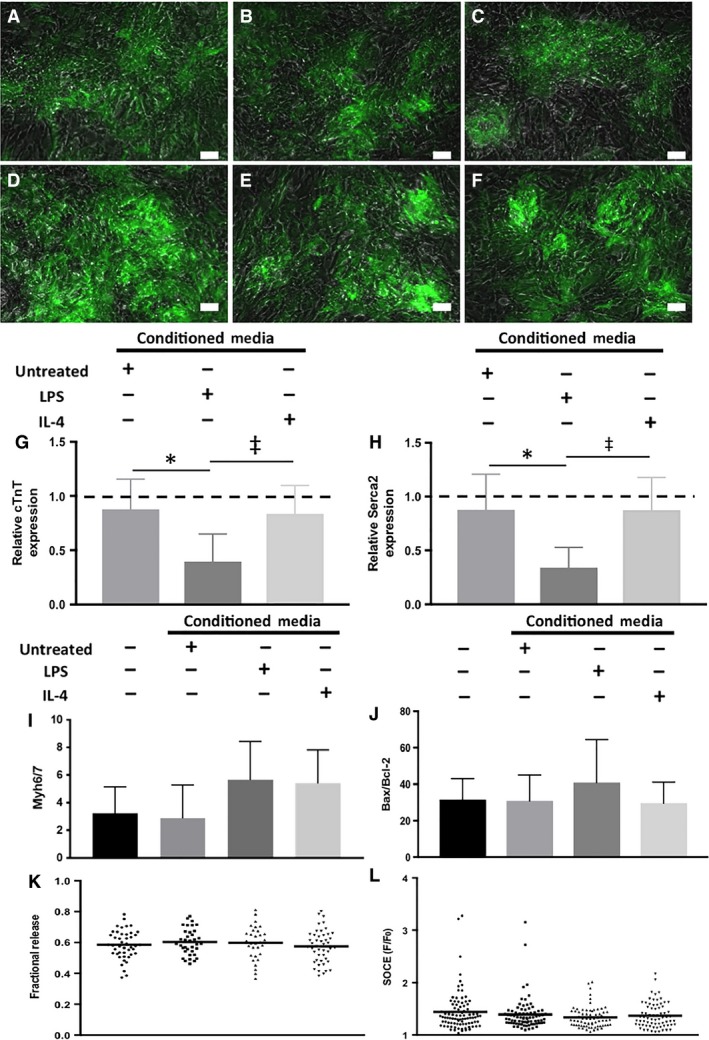
Expression of cTnT‐eGFP (green) by mES‐CM after 72 h in (A) monoculture, (B) with added LPS, (C) with added IL‐4 or in culture with conditioned medium from (D) untreated, (E) LPS‐treated or (F) IL‐4‐treated macrophages (scale bars = 50 *μ*m). RT‐PCR results demonstrate expression of cardiac‐specific markers such as (G) cTnT (*n* = 7 for all, One‐way ANOVA and Tukey, *^,‡^ *P* < 0.05) and (H) Serca2 (*n* = 7 for all, One‐way ANOVA and Tukey, *^,‡^ *P* < 0.05) as well as the ratio of (I) Myh6/7 (*n* = 7 for all, One‐way ANOVA) and apoptosis markers (J) BAX/BCL‐2. (Untreated *n* = 6, *n* = 7 for the rest, Kruskal–Wallis) (K) Ca^2+^ fractional release was calculated as the ratio of paced and caffeine‐induced Ca^2+^ transient amplitudes (*N* = 4 independent trials, mES‐CM Monoculture *n* = 45, Untreated Conditioned Medium *n* = 36, LPS‐treated Conditioned Medium *n* = 31, IL‐4‐treated Conditioned Medium *n* = 46, One‐way ANOVA) and (L) SOCE was measured after treatment with caffeine and CPA (*N* = 3 independent trials, mES‐CM Monoculture *n* = 89, Untreated Conditioned Medium *n* = 80, LPS‐treated Conditioned Medium *n* = 70, IL‐4‐Treated Conditioned Medium *n* = 71, Kruskal–Wallis).

Figure 3A–F display representative images of mES‐CM in all experimental groups expressing cTNT‐eGFP after 3 days of culture. RT‐PCR results demonstrated a significant downregulation of cTnT (Fig. 3G) and Serca2 (Fig. 3H) in mES‐CM cultured in LPS‐treated macrophage‐conditioned medium compared to mES‐CM cultured in either untreated or IL‐4‐treated macrophage‐conditioned medium (*P* < 0.05). However, no significant differences were found in the ratio of Myh6/7 with culture in macrophage‐conditioned medium (Fig. 3I). The ratio of Bax/BCL‐2, which is a classical apoptosis marker, also remained unchanged in mES‐CM cultured in macrophage‐conditioned medium (Fig. 3J).

Calcium‐handling properties of mES‐CM were also explored after 72 h. MES‐CM cultured in LPS‐treated macrophage‐conditioned medium exhibited significantly lower paced and caffeine‐induced transient amplitude compared to mES‐CM in IL‐4‐treated macrophage‐conditioned medium (*P* < 0.05, Table 2) and significantly lower paced transient amplitude compared to mES‐CM in untreated macrophage‐ conditioned medium (*P* < 0.05, Table 2). LPS‐treated macrophage‐conditioned medium also induced significantly longer caffeine‐induced transient *T*_50_ in mES‐CM compared to monoculture (*P* < 0.05, Table 2). Although paced and caffeine‐induced transient morphology differed among groups, fractional release, which is the measure of calcium‐handling efficiency, remained similar among mES‐CM in all culture conditions (Fig. 3K).

**Table 2:** Characteristics of mES‐CM Calcium‐Handling in Indirect Model

	mES‐CM	Conditioned medium		
		Untreated	LPS‐treated	IL‐4‐treated
*N*,* n*	4, 45	4, 36	4, 31	4, 46
Amp (*F*/*F*_0_)	1.51 ± 0.18	1.53 ± 0.21	1.41 ± 0.16‡ ^,^ #	1.52 ± 0.18
*T*_50_ (msec)	159.6 ± 84.1	181.3 ± 107.2	180.0 ± 85.1	157.8 ± 81.8
CI Amp (*F*/*F*_0_)	2.61 ± 0.32	2.55 ± 0.34	2.41 ± 0.40#	2.71 ± 0.41
CI *T*_50_ (msec)	813.5 ± 367.1	942.0 ± 245.6	1297.6 ± 816.2*	925.7 ± 286.2
*N*,* n*	3, 89	3, 80	3, 70	3, 71
Baseline (*F*/*F*_0_)	1.09 ± 0.03	1.12 ± 0.08	1.10 ± 0.07	1.12 ± 0.07*
SR Stores (*F*/*F*_0_)	2.19 ± 0.48	1.98 ± 0.33* ^,^ #	2.16 ± 0.42‡	2.19 ± 0.55

Values are mean ± SD. *N*, independent trials; *n*, cell number; Amp, paced transient amplitude; *T*_50_, paced transient *T*_50_; CI Amp, caffeine‐induced transient amplitude; CI *T*_50_, caffeine‐induced transient *T*_50_; baseline, baseline Ca^2+^ entry; SR stores, sarcoplasmic reticulum Ca^2+^ stores; One‐way ANOVA and Tukey or Kruskal–Wallis and Games‐Howell; *P < 0.05 versus monoculture; ^‡^ *P* < 0.05 versus untreated; ^#^ *P* < 0.05 versus IL‐4‐treated.

MES‐CM cultured in IL‐4‐treated macrophage‐conditioned medium exhibited significantly higher baseline Ca^2+^ entry, or the amount of Ca^2+^ that enters the cell when the extracellular fluid Ca^2+^ concentration changes from 0 mmol/L to 1 mmol/L, compared to mES‐CM in monoculture (*P* < 0.05, Table 2). MES‐CM in untreated macrophage‐conditioned medium culture exhibited significantly lower total sarcoplasmic reticulum (SR) Ca^2+^ stores compared to mES‐CM in monoculture as well as LPS‐treated and IL‐4‐treated macrophage‐conditioned medium culture (*P* < 0.05, Table 2). However, no significant differences in SOCE response were found among mES‐CM in conditioned medium culture (Fig. 3L).

### Direct co‐culture myocardial inflammation model: macrophage characterization

**Figure 4:**
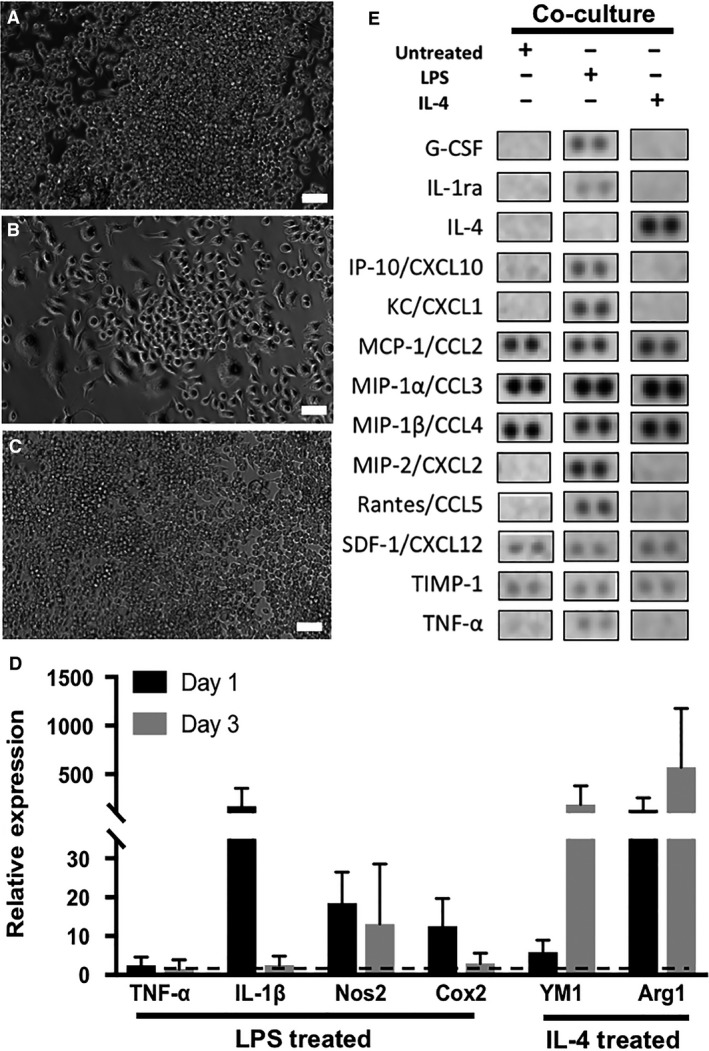
(A) Untreated, (B) LPS‐treated and (C) IL‐4‐treated macrophages in co‐culture with mES‐CM (scale bars = 50 *μ*m). (D) Gene expression profiles of LPS and IL‐4‐treated macrophages in co‐culture with mES‐CM (Day 1 *n* = 3, Day 3 *n* = 5, Independent Samples *T*‐test or Mann–Whitney Test). (E) Cytokine array results showing cytokines present in direct co‐culture conditions, *n* = 1.

Macrophages were initially seeded in isolation in the outer edge of the dishes, however, due to their highly proliferative nature; some macrophages migrated toward mES‐CM in the center of the dish to form direct cell–cell interactions with mES‐CM. Even in this direct co‐culture with mES‐CM, macrophages retained similar morphology as compared to those in monoculture after 3 days (Fig. 4). Both untreated (Fig. 4A) and IL‐4‐treated (Fig. 4C) macrophages remained rounded, while LPS‐treated macrophages (Fig. 4B) were more spread out with projections.

The expression of classical pro‐inflammatory genes such as IL‐1*β* and Nos2 was upregulated in LPS‐treated macrophages in co‐culture with mES‐CM compared to untreated. Similarly, upregulation of anti‐inflammatory genes, such as YM1 and Arg1, was present in IL‐4‐treated macrophages in co‐culture with mES‐CM up to 3 days (Fig. 4D). Pro‐inflammatory cytokines, such as G‐CSF, IL‐1ra, IP‐10, Rantes, MIP‐2 and TNF‐*α* were present in co‐culture of mES‐CM and LPS‐treated macrophages after 3 days (Fig. 4E). However, downregulation of pro‐inflammatory cytokine secretion as well as upregulation of IL‐4 secretion was seen in co‐culture of mES‐CM and IL‐4‐treated macrophages by day 3 (Fig. 4E). The expression of MCP‐1, MIP‐1*α*, MIP‐1*β*, stromal‐derived factor 1 (SDF‐1) and tissue inhibitor of metalloproteinase 1 (TIMP‐1) was similar in all groups (Fig. 4E).

### Direct co‐culture myocardial inflammation model: mES‐CM characterization

**Figure 5:**
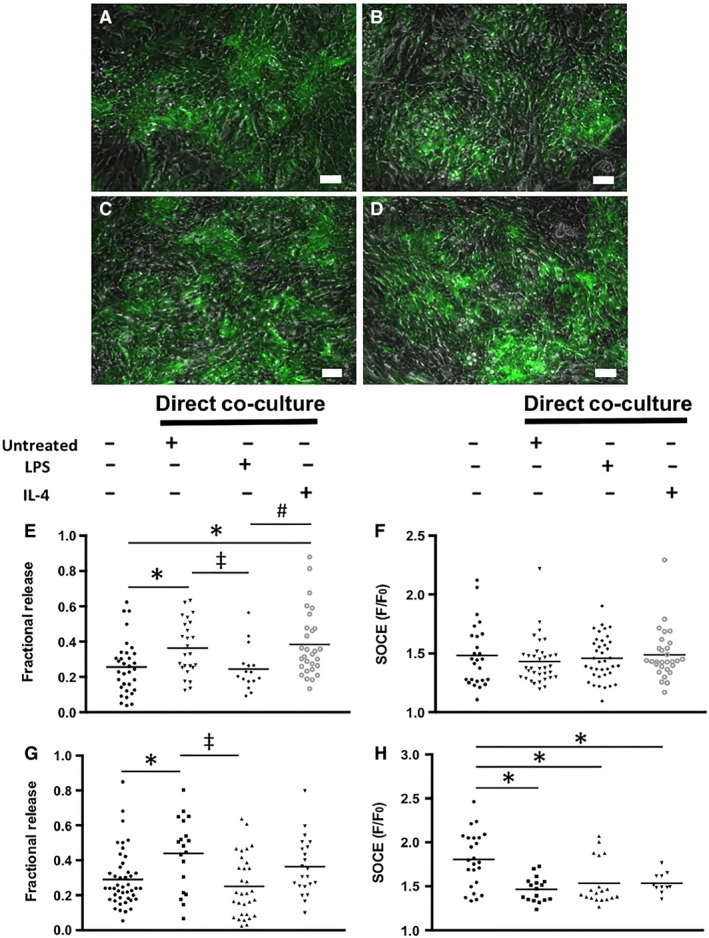
Expression of cTnT‐eGFP in mES‐CM in (A) monoculture and in co‐culture with (B) untreated, (C) LPS‐treated or (D) IL‐4‐treated macrophages (Scale bars = 50 *μ*m). (E) Ca^2+^ fractional release (*N* = 3 independent trials, mES‐CM Monoculture *n* = 32, Untreated Co‐culture *n* = 25, LPS‐treated Co‐culture *n* = 14, IL‐4‐Treated Co‐culture *n* = 27, One‐way ANOVA and Tukey, *^,‡,#^ *P* < 0.05) and (F) SOCE (*N* = 3 independent trials, mES‐CM Monoculture *n* = 26, Untreated Co‐culture *n* = 35, LPS‐treated Co‐culture *n* = 38, IL‐4‐Treated Co‐culture *n* = 29, Kruskal–Wallis) of mES‐CM in monoculture or direct co‐culture with macrophages after 24 h. (G) Ca^2+^ fractional release (*N* = 6 independent trials, mES‐CM Monoculture *n* = 45, Untreated Co‐culture *n* = 19, LPS‐treated Co‐culture *n* = 31, IL‐4‐Treated Co‐culture *n* = 22, Kruskal–Wallis and Games‐Howell, *^,‡^ *P* < 0.05) and (H) SOCE (*N* = 4 independent trials, mES‐CM Monoculture *n* = 24, Untreated Co‐culture *n* = 17, LPS‐treated Co‐culture *n* = 19, IL‐4‐Treated Co‐culture *n* = 11, Kruskal–Wallis and Games‐Howell, *P < 0.05) of mES‐CM in monoculture or direct co‐culture with macrophages after 72 h.

During 3 days of co‐culture with macrophages, similar cTnT‐eGFP expression was observed in mES‐CM independent of macrophage phenotype (Fig. 5A–D). However, even after only 24 h of co‐culture with macrophage subsets, significant changes in calcium‐handling behavior were observed. After 24 h, mES‐CM co‐cultured with IL‐4‐treated macrophages exhibited significantly higher paced transient amplitude compared to mES‐CM cultured in all the other conditions (*P* < 0.05, Table 3) and significantly higher caffeine‐induced transient amplitude compared to mES‐CM in monoculture or untreated macrophage co‐culture (*P* < 0.05, Table 3). No significant differences in paced or caffeine‐induced transient *T*_50_ were found.

**Table 3:** Calcium‐handing properties of mES‐CM in co‐culture

	mES‐CM	Direct Co‐culture		
		Untreated	LPS‐treated	IL‐4‐treated
24 h				
*N*,* n*	3, 33	3, 25	3, 16	3, 29
Amp, *F*/*F*_0_	1.2 ± 0.1	1.31 ± 0.17*	1.29 ± 0.15	1.48 ± 0.19* ^,^ ‡ ^,^ §
*T*_50_, msec	133.6 ± 59.0	138.0 ± 52.5	225.2 ± 124.8	188.9 ± 105.5
CI Amp, *F*/*F*_0_	1.9 ± 0.4	1.94 ± 0.58	2.24 ± 0.63	2.45 ± 0.58* ^,^ ‡
CI T_50,_ msec	1817.5 ± 1618.9	1377.6 ± 655.8	1515.7 ± 891.8	1193.9 ± 602.0
72 h				
*N*,* n*	6, 45	6, 19	6, 31	6, 22
Amp, *F*/*F*_0_	1.3 ± 0.2‡	1.63 ± 0.37	1.24 ± 0.15‡	1.36 ± 0.14‡ ^,^ §
*T*_50_, msec	136.9 ± 91.6	180.1 ± 70.3	129.1 ± 47.6‡	169 ± 88.7
CI Amp, *F*/*F*_0_	2.2 ± 0.4	2.36 ± 0.52	2.11 ± 0.46	2.07 ± 0.35
CI *T*_50,_ msec	1335.6 ± 762.8‡	914.1 ± 427.7	1376.4 ± 693.2‡	1025.4 ± 287.0

Values are mean ± SD. *N*, independent trials; *n*, cell number; Amp, paced transient amplitude; *T*_50_, paced transient *T*_50_; CI Amp, caffeine‐induced transient amplitude; CI *T*_50_, caffeine‐induced transient *T*_50_; One‐way ANOVA and Tukey or Kruskal–Wallis and Games‐Howell; *P < 0.05 versus monoculture; ^‡^ *P* < 0.05 versus untreated; ^§^ *P* < 0.05 versus LPS‐treated.

After 3 days, mES‐CM co‐cultured with IL‐4‐treated macrophages exhibited significantly higher paced transient amplitude compared to mES‐CM in LPS‐treated macrophage co‐culture (*P* < 0.05, Table 3). MES‐CM co‐cultured with untreated macrophages also exhibited significantly higher paced transient amplitude than mES‐CM in all other conditions (*P* < 0.05, Table 3) and significantly longer paced transient *T*_50_ compared to co‐culture with LPS‐treated macrophages (*P* < 0.05, Table 3). However, mES‐CM in co‐culture with untreated macrophages also exhibited significantly shorter caffeine‐induced transient *T*_50_ compared to mES‐CM in monoculture and co‐culture with LPS‐treated macrophages (*P* < 0.05, Table 3). Additionally, mES‐CM co‐cultured with untreated and IL‐4‐treated macrophages exhibited significantly higher fractional release compared to mES‐CM in monoculture or LPS‐treated macrophage co‐culture after 24 h (*P* < 0.05, Fig. 5E). MES‐CM co‐cultured with untreated macrophages maintained significantly higher fractional release compared to mES‐CM in monoculture and LPS‐treated macrophage co‐culture up to 3 days (*P* < 0.05, Fig. 5G).

SOCE‐related properties were also measured in mES‐CM in co‐culture with macrophage subsets (Table 4). After 24 h, mES‐CM co‐cultured with LPS‐treated macrophages exhibited significantly higher baseline Ca^2+^ entry than mES‐CM in co‐culture with untreated macrophages (*P* < 0.05) and significantly higher SR Ca^2+^ stores than all other groups (*P* < 0.05). After 3 days, no significant differences in baseline Ca^2+^ entry were found but mES‐CM co‐culture with untreated macrophages exhibited significantly lower SR Ca^2+^ stores compared to monoculture (*P* < 0.05). While no changes in SOCE were observed after the first 24 h of co‐culture (Fig. 5F), significantly depressed SOCE response was demonstrated after 3 days of co‐culture with macrophages independent of their subtypes (*P* < 0.05, Fig. 5H).

**Table 4:** Calcium‐Handling Properties Related to SOCE of mES‐CM in Co‐culture

	mES‐CM	Direct Co‐culture		
		Untreated	LPS‐treated	IL‐4‐treated
24 h				
*N*,* n*	3, ≥23	3, ≥34	3, ≥36	3, ≥29
Baseline, *F*/*F*_0_	1.11 ± 0.05	1.10 ± 0.04	1.14 ± 0.06‡	1.13 ± 0.06
SR stores, *F*/*F*_0_	2.07 ± 0.46	2.03 ± 0.45	2.39 ± 0.36* ^,^ ‡ ^,^ #	2.13 ± 0.42
72 h				
*N*,* n*	4, 24	4, ≥16	4, 19	4, ≥10
Baseline, *F*/*F*_0_	1.18 ± 0.08	1.19 ± 0.09	1.22 ± 0.17	1.13 ± 0.03
SR Stores, *F*/*F*_0_	2.18 ± 0.36	1.82 ± 0.37*	1.91 ± 0.46	2.02 ± 0.27

Values are mean ± SD. *N*, independent trials; *n*, cell number; Baseline, Baseline Ca^2+^ Entry; SR stores, sarcoplasmic reticulum Ca^2+^ stores; Kruskal–Wallis and Games‐Howell; *P < 0.05 versus monoculture; ^‡^ *P* < 0.05 versus untreated; ^#^ *P* < 0.05 versus IL‐4‐treated.

### Matricellular protein expression

**Figure 6:**
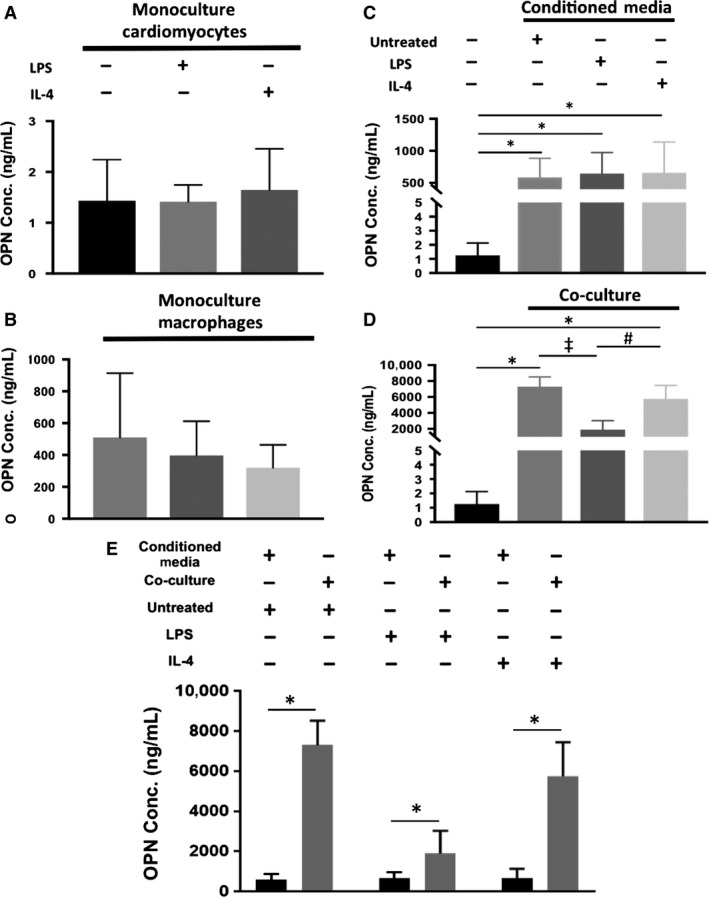
OPN secretion by monoculture (A) mES‐CM after 3 days (Untreated *n* = 7, LPS‐treated *n* = 3, IL‐4‐Treated *n* = 3, One‐way ANOVA) and (B) macrophages after 24 h (Untreated *n* = 4, LPS‐treated *n* = 4, IL‐4‐Treated *n* = 3, One‐way ANOVA). (C) OPN secretion in monoculture and in conditioned medium cultures measured after 3 days (mES‐CM Monoculture *n* = 7, Untreated Conditioned Medium *n* = 5, LPS‐treated Conditioned Medium *n* = 5, IL‐4‐Treated Conditioned Medium *n* = 5, One‐way ANOVA and Tukey, *P < 0.05). (D) OPN secretion in direct co‐culture groups compared to monoculture mES‐CM after 3 days (mES‐CM Monoculture *n* = 7, Untreated Co‐culture *n* = 4, LPS‐treated Co‐culture *n* = 4, IL‐4‐Treated Co‐culture *n* = 4, One‐way ANOVA with Brown‐Forsyth correction and Tukey, *^,‡,#^ *P* < 0.05). (E) OPN secretion in direct co‐culture groups compared to corresponding conditioned medium groups. (Direct Co‐culture *n* = 4, Conditioned Medium *n* = 5, Independent Samples T‐test, *P < 0.05)

OPN secretion in mES‐CM groups was less than 3 ng/mL over 3 days (Fig. 6A) while all monoculture macrophage populations secreted over 200 ng/mL OPN after only 24 h (Fig. 6B). Significantly higher levels of OPN were detected in macrophage‐conditioned medium culture compared to mES‐CM in monoculture after 3 days (*P* < 0.05, Fig. 6C). Additionally, over a thousand fold OPN was detected in all co‐culture conditions compared to mES‐CM in monoculture (Fig. 6D). This was significantly higher than the corresponding conditioned medium groups (*P* < 0.05, Fig. 6E).

**Figure 7:**
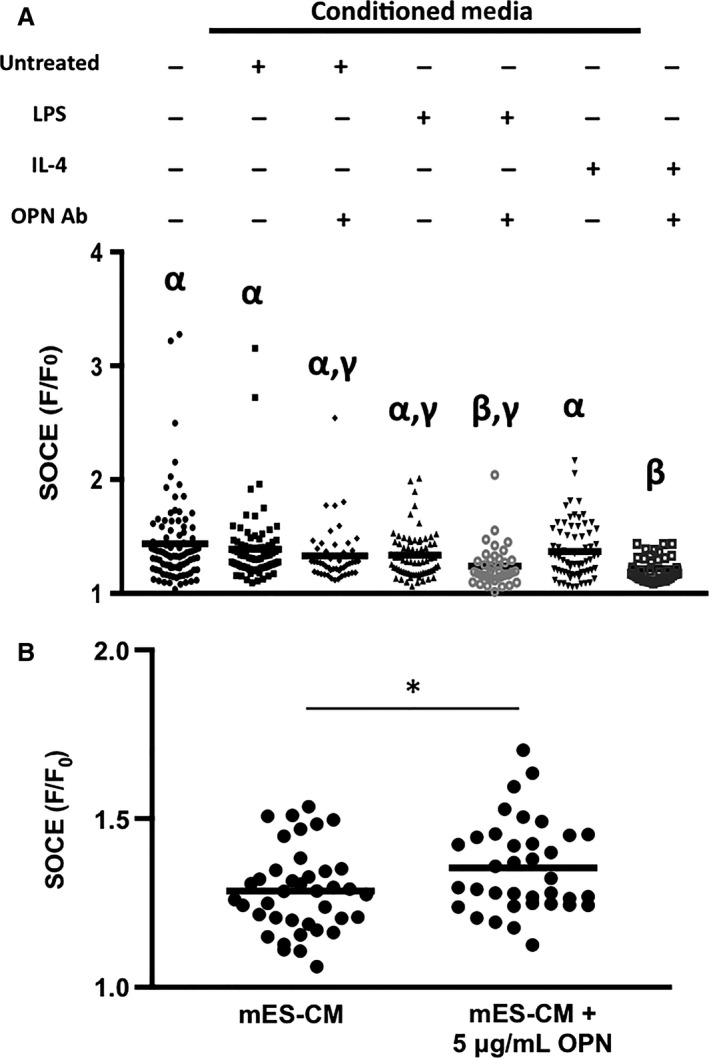
(A) SOCE response of mES‐CM in conditioned medium culture with or without OPN inhibition after 3 days (*N* = 5 independent trials, mES‐CM Monoculture *n* = 89, Untreated Conditioned Medium *n* = 80, Untreated Conditioned Medium + OPN Ab *n* = 48, LPS‐treated Conditioned Medium *n* = 70, LPS‐treated Conditioned Medium + OPN Ab *n* = 38, IL‐4‐Treated Conditioned Medium *n* = 71, IL‐4‐Treated Conditioned Medium + OPN Ab *n* = 8 = 39, Kruskal–Wallis and Games‐Howell, different Greek letters indicate significant differences between the groups, *P* < 0.05). (B) SOCE response in mES‐CM with the addition of recombinant OPN after 3 days. (*N* = 3 independent trials, mES‐CM *n* = 39, mES‐CM + OPN *n* = 36, Independent Samples *T*‐test, *P < 0.05).

When OPN was neutralized using OPN‐specific antibody, significant differences were found in SOCE response of mES‐CM. Specifically, mES‐CM cultured in IL‐4‐treated macrophage‐conditioned medium exhibited the lowest SOCE response with OPN inhibition, significantly lower than that of mES‐CM in all other conditions without OPN inhibition (*P* < 0.05, Fig. 7A). MES‐CM cultured in LPS‐treated macrophage‐conditioned medium with OPN inhibition also exhibited significantly lower SOCE than mES‐CM in monoculture as well as in untreated and IL‐4‐treated macrophage‐conditioned medium without OPN inhibition (*P* < 0.05, Fig. 7A). Additionally, when 5 *μ*g/mL of recombinant OPN was added to mES‐CM in monoculture, SOCE response was significantly increased (*P* < 0.05, Fig. 7B).

## Discussion

While macrophages have emerged as attractive effectors in tissue injury and repair, the contribution of specific macrophage subsets on cardiac cell function and survival is not fully understood (Honold and Nahrendorf 2018). A better understanding of the role of myocardial inflammation post‐MI is especially important for stem cell‐based therapy, as most of the transplanted cells do not endure the inflammatory response and functionally couple with the resident cells in an ischemic environment. However, there are currently no appropriate in vitro experimental models to study the role of myocardial inflammation and macrophages, as existing models focus almost exclusively on mimicking the healthy cardiac microenvironment with the goal of providing a living surgical replacement. Thus, in this study, both an indirect and direct myocardial inflammation model was created to better understand how distinct macrophage subsets affect pluripotent stem cell‐derived cardiomyocyte phenotype and function. These models provided a simple and controlled in vitro platform, which allows convenient evaluation of each cell type individually. This study focuses on the effect of macrophages on calcium‐handling function of cardiomyocytes and its relationship with matricellular proteins to identify a potential mechanism through which macrophages and cardiomyocytes interact post‐MI.

Macrophages of two different subsets were successfully derived as confirmed by their gene expression and cytokine secretion. Macrophages with either M1 and M2 phenotype were used to mimic two distinct phases of inflammatory cells infiltrating and peaking at 3 and 7 days post‐MI. Polarized macrophage subsets exhibited distinct morphologies with the pro‐inflammatory subset exhibiting a more round, spread out morphology consistent with previously described work using bone marrow‐derived macrophages (McWhorter et al. 2013). LPS‐treated macrophages expressed known sets of pro‐inflammatory makers such as TNF‐*α*, IL‐1*β*, Nos2, and Cox2 (Martinez and Gordon 2014). IL‐4‐treated macrophages, expressed significantly higher levels of characteristic anti‐inflammatory markers, including YM1 and ARG1 (Martinez and Gordon 2014), with significantly lower levels of pro‐inflammatory markers as expected. Moreover, LPS treatment upregulated pro‐inflammatory cytokine secretion after 24 h consistent with previously described studies using bone marrow‐derived macrophages and RAW264.7 cells (Mosser and Edwards 2008; Chung et al. 2009; Melton et al. 2015; Ai et al. 2018). Upregulated pro‐inflammatory cytokines included MCP‐1, MIP‐1*α*, and RANTES, which match with the cytokines found in serum of patients up to 7 days post‐MI (Kobusiak‐Prokopowicz et al. 2005), suggesting the translational potential of the model. Moreover, IL‐4 treatment induced a downregulation of pro‐inflammatory cytokine secretion as well as upregulation of IL‐4 secretion as previously demonstrated (Choi and Reiser 1998).

To explore the effects of macrophage‐derived factors on cardiomyocyte function, conditioned medium from polarized macrophage subsets was first used to culture mES‐CM. Our results suggest that differentiation and maturation of mES‐CM was not affected by macrophage‐secreted factors as evidenced by similar expression of Myh6/7 ratio in all conditioned medium groups. Moreover, similar BAX and BCL‐2 expression suggested that macrophage‐derived factors do not affect mES‐CM apoptosis in contrast to a recent study which reported increased cardiomyocyte apoptosis when in indirect culture with pro‐inflammatory macrophages (Ai et al. 2018). However, while LPS‐treated macrophage‐conditioned medium culture elicited significant downregulation of cardiac‐specific marker cTnT in mES‐CM, IL‐4‐treated conditioned medium culture demonstrated maintenance of cTnT expression suggesting there could be recovery of expression in an anti‐inflammatory microenvironment. Serca2, which is the principle regulator of cytoplasmic Ca^2+^ efflux (Bers 2002) was also significantly downregulated in mES‐CM cultured in LPS‐treated macrophage‐conditioned medium. Downregulation of Serca2 suggests that a prolonged or exaggerated pro‐inflammatory environment could be one of the contributing factors in the dysregulation of calcium‐handling post‐MI (Currie and Smith 1999). Consistent with a previous study demonstrating that Serca2 upregulation post‐MI may aid in preservation of ventricular function (Fernandes et al. 2015), our study suggests that timely resolution of inflammation may improve ventricular function through increased Serca2 expression.

Another calcium‐handling mechanism known to be upregulated in response to stress or inflammation is SOCE (Kojima et al. 2012; Zheng et al. 2017; Wen et al. 2018). Traditionally found in non‐electroactive cell types, SOCE is associated with changes in SR Ca^2+^ stores. While there exists some debate over whether SOCE is present in healthy adult cardiac tissue, it has recently been identified in cardiomyocytes during the embryonic and neonatal phase of development (Luo et al. 2012). In our study, mES‐CM demonstrate SOCE response as has been previously demonstrated in embryonic stem cell‐derived cardiomyocytes (Youm 2016). However, macrophage‐conditioned medium did not cause significant changes in SOCE response of mES‐CM. Since mES‐CM demonstrate SOCE response, it is plausible that its intrinsic SOCE response masked any further changes in SOCE response below the threshold.

However, in the direct co‐culture model, significant changes in calcium‐handling function in mES‐CM were demonstrated suggesting direct cell–cell interaction was necessary to elicit changes in cardiomyocyte function. Significant upregulation of Ca^2+^ fractional release as well as downregulation of SOCE response of mES‐CM in co‐culture with macrophages suggest that macrophages may be contributing to the Ca^2+^ handling function of mES‐CM specifically. Since upregulation of SOCE has been associated with the development of cardiac hypertrophy (Hulot et al. 2011; Luo et al. 2012; Zheng et al. 2017), downregulation of SOCE response as seen in direct co‐culture suggests that direct interaction with macrophages could play a role in preventing the development of hypertrophy in cardiomyocytes.

Similar to SOCE, OPN is also associated with the development of cardiac hypertrophy and the regulation of Ca^2+^ homeostasis (Murry et al. 1994; Graf et al. 1997; Stawowy et al. 2002; Xie et al. 2004) despite playing a crucial role in the healing process immediately after MI (Trueblood et al. 2001). Our results confirmed that OPN was mostly secreted by macrophages independent of their phenotype. The OPN level in all macrophage‐conditioned medium groups was within a range of serum OPN concentration detected in patients within 12 h of MI onset (Suezawa et al. 2005). Thus, no significant functional changes correlated with OPN in our experimental groups are likely due to OPN concentration threshold not being reached. The inhibition of OPN, however, elicited significant changes in mES‐CM SOCE response, especially in culture with LPS‐ and IL‐4‐treated macrophage‐conditioned medium suggesting a link between OPN and SOCE response in mES‐CM. Further, the addition of recombinant OPN at an elevated concentration similar to plasma OPN concentrations in patients from day 3 to day 7 post‐MI (Suezawa et al. 2005), induced significant upregulation of SOCE in mES‐CM. This further confirms that OPN could play an important role in the disruption of Ca^2+^ homeostasis through the upregulation of SOCE potentially leading to long‐term pathological hypertrophy development if not regulated properly.

Interestingly, this upregulation of SOCE by significantly elevated levels of OPN was completely reversed when direct contact between mES‐CM and macrophages was present. This suggests that while OPN may upregulate SOCE, direct contact with macrophages significantly suppresses SOCE in mES‐CM. Since SOCE is associated with down‐stream Ca^2+^ dysregulation and cardiac hypertrophy, direct contact with macrophages promoting SOCE downregulation in cardiomyocytes may be beneficial in the long term. Hulsmans et al. demonstrated direct electrical coupling of macrophages and cardiomyocytes through expression of the photoactivatable channelrhodopsin 2 (ChR2) in macrophages, which is known to be permeable to Ca^2+^ (Nagel et al. 2003). With ChR2 light activation, a flood of cations into macrophages caused depolarization, which affected cardiomyocyte electrophysiological properties such as resting membrane potential (Hulsmans et al. 2017). Therefore, the coupling between macrophages and cardiomyocytes causing significant changes in calcium‐handling behavior demonstrated here could be related to the previously reported changes in electrophysiological properties of macrophage‐coupled cardiomyocytes in vivo. Future studies are required to fully understand the complex interplay between coupled macrophages and cardiomyocytes, specifically regarding Ca^2+^ currents.

Together, this study demonstrated for the first time, novel in vitro myocardial inflammation models dedicated to understanding the effects of activated macrophage subsets on cardiomyocyte calcium‐handling function and its relationship with macrophage‐secreted matricellular proteins. These in vitro models provide a controlled platform in which each cell type can be easily seeded and individually evaluated. This study can be extended to examine the interactions of macrophages with human‐derived cells or mature adult cardiomyocytes. Moreover, the findings from the in vitro model can be validated in in vivo animal models, and findings from in vitro studies can provide insights in predicting and interpreting otherwise complex combinations of factors involved in in vivo studies.

## Conflict of Interest

No conflicts of interest declared.
